# Hybrid Lipid Nanoformulations for Hepatoma Therapy: Sorafenib Loaded Nanoliposomes—A Preliminary Study

**DOI:** 10.3390/nano12162833

**Published:** 2022-08-17

**Authors:** Adrian Bartos, Ioana Iancu, Lidia Ciobanu, Anca Onaciu, Cristian Moldovan, Alin Moldovan, Radu Cristian Moldovan, Adrian Bogdan Tigu, Gabriela Fabiola Stiufiuc, Valentin Toma, Cornel Iancu, Nadim Al Hajjar, Rares Ionut Stiufiuc

**Affiliations:** 1 Department of Surgery, Regional Institute of Gastroenterology and Hepatology, 400162 Cluj-Napoca, Romania; 2 Department of Surgery, “Iuliu Hatieganu” University of Medicine and Pharmacy, 400337 Cluj-Napoca, Romania; 3 Department of Surgery, Medicover Hospital, 407062 Cluj-Napoca, Romania; 4 MedFuture—Research Center for Advanced Medicine, “Iuliu Hatieganu” University of Medicine and Pharmacy, 400337 Cluj-Napoca, Romania; 5 Department of Pharmaceutical Physics-Biophysics, “Iuliu Hațieganu” University of Medicine and Pharmacy, 400349 Cluj-Napoca, Romania; 6 Faculty of Physics, “Babes Bolyai” University, 400084 Cluj-Napoca, Romania

**Keywords:** hybrid liposomal nanoformulation, hepatoma therapy, Raman analysis

## Abstract

Sorafenib is a multikinase inhibitor that has received increasing attention due to its high efficacy in hepatocellular carcinoma treatment. However, its poor pharmacokinetic properties (limited water solubility, rapid elimination, and metabolism) still represent major bottlenecks that need to be overcome in order to improve Sorafenib’s clinical application. In this paper, we propose a nanotechnology-based hybrid formulation that has the potential to overcome these challenges: sorafenib-loaded nanoliposomes. Sorafenib molecules have been incorporated into the hydrophobic lipidic bilayer during the synthesis process of nanoliposomes using an original procedure developed in our laboratory and, to the best of our knowledge, this is the first paper reporting this type of analysis. The liposomal hybrid formulations have been characterized by transmission electron microscopy (TEM), dynamic light scattering (DLS), and nanoparticle tracking analysis (NTA) that provided useful information concerning their shape, size, zeta-potential, and concentration. The therapeutic efficacy of the nanohybrids has been evaluated on a normal cell line (LX2) and two hepatocarcinoma cell lines, SK-HEP-1 and HepG2, respectively.

## 1. Introduction

Hepatocellular carcinoma (HCC) is the most prevalent primary liver cancer and a leading cause of cancer-related deaths worldwide [1,2]. Early-stage tumors benefit from curative techniques, such as resection, liver transplantation, or local ablation. For intermediate-stage HCC, chemoembolization showed survival benefits. Among the targeted therapies, sorafenib was the first systemic drug that showed efficacy in advanced HCC. Sorafenib has represented the standard of care as first-line therapy for over 10 years for advanced HCC [3]. Nowadays, several other drugs have been approved: lenvatinib as first-line along with regorafenib, carbozantinib, and recombinant immunoglobulin G1 (IgG1) and monoclonal antibody ramucirumab as second-line post-sorafenib [3,4,5,6,7,8,9]. On the other hand, the combination of atezolizumab and bevacizumab significantly improved the overall and progression-free survival outcomes compared to sorafenib, in unresectable or metastatic HCC [10,11].

The efficacy of these drugs in HCC is frequently limited by the liver functional re-serve. Many patients present cirrhotic complications (variceal bleeding, hepatic encephalopathy, spontaneous bacterial peritonitis, and hepatorenal syndrome). A more tailored therapy is warranted in cases with limited liver functional reserve. Local ablative techniques performed under imaging guidance combined with a targeted therapy towards the tumoral cell might improve the beneficial effects of these drugs in locally advanced HCC cases.

A possible approach that can help in overcoming these major drawbacks is based on nanotechnology, especially in the case of the molecular targeting agents used for the treatment of unresectable hepatocarcinoma, such as sorafenib [12]. The scientific literature reports on different nanoformulations that have been used for specific targets and enhancements of pharmaceutical properties, and/or codelivery of multiple drugs [12]. Based on the unique properties of nanomaterials, our research group proposed different types of hybrid nanostructures that have been successfully tested for cancer therapy [13,14] and/or other biomedical/pharmaceutical applications [15]. Nevertheless, other nanohybrids have also been employed for the visualization of drug delivery sites on computerized tomography (CT) scans or ultrasound imaging [3]. Despite these promising features, their therapeutic effects are still limited, partly by inadequate delivery due to the heterogeneous enhanced permeability and retention (EPR) effect, dense extracellular matrix, and high interstitial fluid pressure in tumors [16]. To overcome these challenges, some previous studies demonstrated that ultrasound in combination with microbubbles improved the delivery, local distribution, and therapeutic efficacy of pristine nanoparticles and/or drug-loaded nanoparticles in tumors [16,17]. Ultrasound-mediated effects, also known as sonopermeation, might increase vascular permeability and thereby increase extravasation and potentially improve penetration through the extracellular matrix, which could result in enhanced accumulation and distribution of nanoparticles and drugs in tumor tissues [16,18].

Among all the nanostructures that have been synthesized and tested for medical ap-plications, liposomes are a special class of nanoparticles that might be useful in HCC treatment considering their capacity to encapsulate cytotoxic drugs and their ultrasound-mediated delivery potential [19].

The encapsulation of cytotoxic drugs within liposomes enhances the pharmacokinetics of the cytotoxic agent and allows its passive accumulation within tumors. Liposomes consist of a single or multiple concentric lipid bilayers encapsulating an aqueous core [20,21]. Initially, they included natural lipids [22,23], but nowadays they consist of natural and/or synthetic lipids and surfactants, similar to those present in human cells. One of their most important properties is the capability of entrapping both lipophilic agents (in the lipid membrane) and hydrophilic ones (in the aqueous core), respectively [20,24,25].

Nowadays, several liposomes-based nanoformulations are already used in clinical practice for the treatment of different cancers: Doxil (liposomal doxorubicin), onivyde (liposomal irinotecan), vyxeos (liposomal cytarabine and daunorubicin), but in the case of HCC, such a nanoformulation has not yet been approved. However, there are a few studies that aim to develop promising nanoliposomal systems for chemotherapeutic efficient delivery. The Pan Li group reported the in vivo use of doxorubicin-loaded liposomes on an HCC mouse model. They observed that by combining this therapy with ultrasound-targeted microbubble destruction, an increased antitumor effect, as compared to the treatment alone, could be achieved [26]. Another strategy, developed by the Ping Liang group, proposed the use of doxorubicin-encapsulated liposomes together with mild microwave ablation, revealing optimistic results for the HCC treatment [27]. Other recent studies focused on codelivery of various drugs for HCC treatment: Aprepitant and curcumin [28], combretastatin A4 phosphate and curcumin [29], cisplatin and curcumin [30], sorafenib and gadolinium [31], sorafenib and doxorubicin [32]. All these studies showed promising results for HCC therapy. On the other hand, the active targeting of HCC cells by nanoparticles functionalized with specific ligands-proteins [33], antibodies [34], or other molecules (glycyrrhetinic acid [28,29], hyaluronic acid [28,35]) has attracted great scientific interest, and the results reported in these studies are also promising.

Such discoveries encouraged our group to investigate the advantages offered by a specific class of nanoparticles (liposomal nanosystems) for HCC treatment. As such, in this study, we aimed to synthesize a hybrid lipid nanoformulation—sorafenib-functionalized nanoliposomes—to characterize them and evaluate the therapeutic effects of these new nanohybrids through in vitro studies on normal and HCC cell cultures.

## 2. Materials and Methods

Materials: 1,2-distearoyl-sn-glycero-3-phosphocholine (DSPC, Avanti Polar Lipids, Alabaster, AL, USA); cholesterol (Chol, Avanti Polar Lipids, Alabaster, AL, USA); 1,2-dipalmitoyl-sn-glycero-3-phospho- (1′-rac-glycerol) (DPPG, Avanti Polar Lipids, Alabaster, AL, USA); chloroform, methanol (Chemical Company, Iasi, Romania); sorafenib (Sigma Aldrich, St. Louis, MI, USA); saline solution (B. Braun Medical, Melzungen, Germany); fetal bovine serum (FBS) (Gibco, Thermo Fischer Scientific, Waltham, MA, USA); and cell culture media: DMEM HG and MEM (Gibco, Thermo Fisher Scientific, Waltham, MA, USA). Supplements: glutamine (Thermo Fischer Scientific, Waltham, MA, USA).

### 2.1. Synthesis of Sorafenib Functionalized Liposomes

The liposomes were synthesized using a rotary evaporator (Heidolph Instruments, Schwalbach, Germany) with a solvent evaporation method.

The first step in the liposome synthesis was the formation of a lipid mixture composed of DSPC: Chol: DPPG 12: 1: 6 (5.1 mg) solubilized in a mixture of chloroform: methanol 2: 1 (having a volume of 3 mL). Then, the solvent evaporation protocol, and hydration with a saline solution (volume 5 mL) was conducted using Heidolph Precision Rotary Evaporator equipment.

The formation of the lipid film was performed in a water bath at 40 °C, with 400 mbar pressure, 80 rpm, 10 °C cooling temperature for 2 h. For the hydration stage, 5 mL of 0.9% saline solution was used, at a temperature of 56 °C of the water bath, atmospheric pressure, and 80 rpm for 1 h. The last step was to ultrasonicate the solution for 30 min at a temperature of approximately 50 °C.

In the case of sorafenib-loaded (SOR) liposomes (Lipo_SOR), 0.5 mg of drug was added in the lipid solubilization stage, following the same steps described above.

### 2.2. Characterization of Liposomes

#### 2.2.1. Raman Spectroscopy

Raman analysis of the synthesized liposomes aimed to highlight the organization of lipid molecules in the structure of liposomes, respectively. The identification of experimental data to confirm the functionalization of sorafenib in the lipid bilayer.

For this measurement, a simple “drop coating method” was used with the deposition of ~2 µL of the liposome solution on an aluminum foil surface. After drying the analyte (~30 min at room temperature), the Raman spectra were recorded at a maximum of 50 µm from the outer edge of the dry sample on the support by using an inVia™ confocal Raman microscope (Renishaw, UK).

Three types of samples were measured: blank liposomes (Lipo), Lipo_SOR, and pure SOR, respectively. The 785 nm laser wavelength in the spectral range of 200–3200 cm^−1^ was used as follows:

- Lipo and Lipo_SOR: excitation wavelength 785 nm, power at sample surface 113 mW, objective lens 50×, 60 acquisition points, integration time 40 s (exposure time 10 s and 4 signal accumulations).
- SOR: excitation wavelength 785 nm, power at sample surface 11.3 mW, objective lens 50× lens, 20 acquisition points, integration time 40 s (exposure time 10 s and 4 signal accumulations).

#### 2.2.2. Determination of Concentration, Zeta-Potential and Particle Size of Liposomes

Nanoparticle tracking analysis (NTA, Stabino, UK) method was used for liposome characterization and measurement. The liposome solutions (Lipo and Lipo_SOR) were diluted in ultrapure water to determine their concentration, zeta-potential, and size.

#### 2.2.3. Transmission Electron Microscopy Measurements

For TEM analysis, 200 µL of each liposome solution was mixed with 1200 µL ultrapure water and 1 µL osmium tetroxide 4%. The samples were incubated at 4 °C for 60 min and then centrifuged at 12,000× *g* for 5 min. The supernatant was removed, and the liposomes were resuspended in 200 μL ultrapure water.

5 µL of this liposomal solution was deposited on a TEM grid supporting a film of 400 mesh of carbon. After 2–5 min, the excess liquid was removed with a filter paper, and the sample was allowed to dry at room temperature. TEM images of the liposomes were taken using Hitachi HT 7000 Transmission Electron Microscopy equipment (Hitachi Ltd. Tokyo, Japan), 100 kV, equipped with a high-resolution 8-Megapixel CCD camera.

#### 2.2.4. Sorafenib Encapsulation Efficiency

The concentration of SOR entrapped in liposomes was investigated with the HPLC coupled to UV detection (265 nm) using a Prominence HPLC system (Shimadzu, Kyoto, Japan). The chromatographic conditions were adapted after a method described by Blanchet et al. [36]. In brief, the separation was carried out on a BEH C18 column (2.1 × 100 mm^2^, 1.7 µm particle diameter) (Waters, Milford, MA, USA) thermostated at 40 °C, with mobile phases composed of (A) 20 mM ammonium acetate and (B) acetonitrile, being pumped at 0.3 mL/min. The analyte was eluted using a gradient starting from 60% B (0 min) to 70% B (5 min).

Before analysis, the Lipo_SOR solution was centrifuged at 4 °C for 5 min at 12,000 rpm to pellet the liposomes. Five µL of the resulting supernatant was analyzed in order to determine the concentration of the unencapsulated sorafenib. The concentration has been calculated using a 5-level calibration curve between 10 and 150 µg/mL. All measurements have been performed in triplicate.

Sorafenib encapsulation efficiency—*EE*% was calculated using the following equation:

(1) $$EE\%=\left(\frac{C_{total\;drug}-C_{unencpasulated\;drug}}{C_{total\;drug}}\right)\times 100$$

*C_unencapsulated drug_* represents the concentration of SOR that was free in the media, while *C_total drug_* represents the concentration of SOR added during the liposome synthesis procedure.

### 2.3. In Vitro Cytotoxicity Studies on Cell Cultures

In vitro studies were performed on three hepatic cell lines: a normal human hepatic stellate cell line (LX-2), and two human hepatic adenocarcinoma cell lines (SK-HEP-1 and HepG2).

The LX-2 cell line was cultured in DMEM with 10% FBS, supplemented with 1% glutamine, while HepG2 and SK-HEP-1 cell lines were cultured in MEM with 10% FBS. All cell lines were grown at 37 °C in a CO_2_ atmosphere.

Cytotoxicity studies with sorafenib and synthesized nanoparticles (blank liposomes and sorafenib-encapsulated liposomes) to determine the IC50 concentration were performed at 24 and 48 h.

96-well cell culture plates were used for cell growth (7000 cell/well). At 24 h after culturing the plates, the treatment (SOR/Lipo_SOR) was added at different concentrations in the range of 2.5–20 µM and 1.88–15.08 µM, respectively. In the case of blank liposomes, their concentrations were adapted to the Lipo_SOR doses.

The stock solution of sorafenib was obtained by diluting SOR in DMSO and then the working solutions were prepared by serial dilutions with saline solution. The other two treatments dosees were obtained by serial dilutions with saline solution.

MTT cellular viability assay with an incubation step at 37 °C for 3 h was performed 24 and 48 h after the application of the treatment. The absorbance was measured using the Spark 10M microplate reader (Tecan, Männedorf, Switzerland), and the results were analyzed using GraphPad Prism 6 software.

## 3. Results

### 3.1. Characterization of Liposomes

#### 3.1.1. Evaluation of Sorafenib-Loaded Nanoliposome by Raman Spectroscopy

In order to evaluate the molecular interaction between sorafenib and the lipid molecules composing the liposomal bilayer, we performed Raman measurements on sorafenib, blank liposomes, and sorafenib-loaded nanoliposomes, using a near-infrared (NIR) excitation laser (785 nm).

For each spectrum, the following processing was performed: removal of peaks from the environment, removal and smoothing of noise from spectra, spectrum mediation, removal of fluorescence background, design with an individual marking of peaks in Origin Pro2019, overlapping spectra for the identification of similarities/differences, calculation, and graphical expression according to the number of counts obtained at a certain nominal power of the laser on the sample.

**Figure 1:**
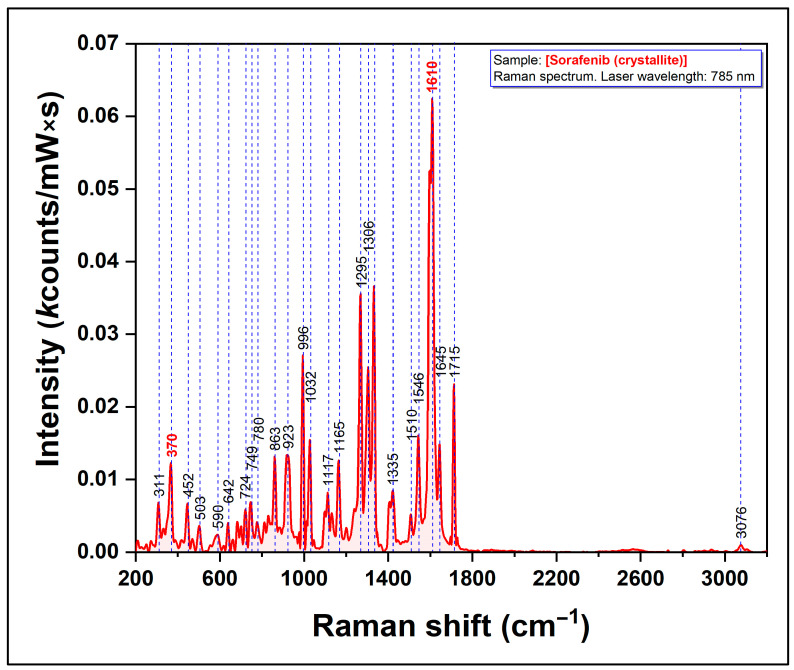
Raman spectrum of pure sorafenib molecules (the excitation laser is 785 nm).

The Raman spectrum of pure SOR molecules (crystallite powder) recorded using a NIR excitation laser (785 nm) is shown in Figure 1.

**Figure 2:**
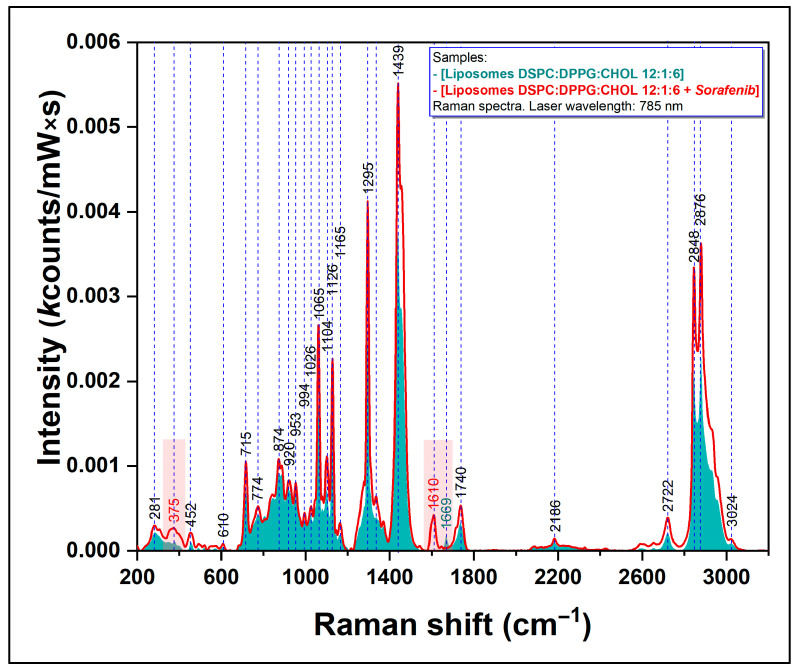
Raman spectra of pure (blue line) and sorafenib-loaded nanoliposomes (red curve) (the laser wavelength is 785 nm).

After the synthesis procedures of Lipo and Lipo_SOR were completed, we performed a Raman characterization of the two analytes, using the same experimental conditions (excitation wavelength, exposure, acquisition time, etc.) as those used in the case of pure SOR. The spectra of blank nanoliposomes (blue spectrum) and of Lipo_SOR (red spectrum) are presented in Figure 2.

Raman spectroscopy was employed as a direct proof of sorafenib’s incorporation into the lipidic bilayer. Therefore, the Raman spectra recorded on pure (unloaded) liposomes were compared with those acquired on Lipo_SOR.

The main vibrational bands of the lipids used for the synthesis of the nanoliposomes are present in our spectra. Their complete assignment was previously reported in a paper published by our research group (Supplementary Materials Table S3, [23]).

#### 3.1.2. Determination of Concentration, Zeta-Potential and Particle Size of Liposomes

Data regarding the concentration, particle size, and zeta-potential of Lipo and Lipo_SOR are presented in Table 1. In the case of pure liposomes, their diameters were approximately 180 nm for ~93% of the population. In the case of Lipo_SOR populations, average sizes between 130 and 320 nm were predominant (~70% of the population).

The liposome’s concentration was ~5 × 10^12^ (NP/mL) (pure liposomes) with respect to ~9 × 10^10^ NP/mL (Lipo_SOR). The zeta-potential values were slightly positive in both cases.

#### 3.1.3. Transmission Electron Microscopy (TEM) Analysis

**Figure 3:**
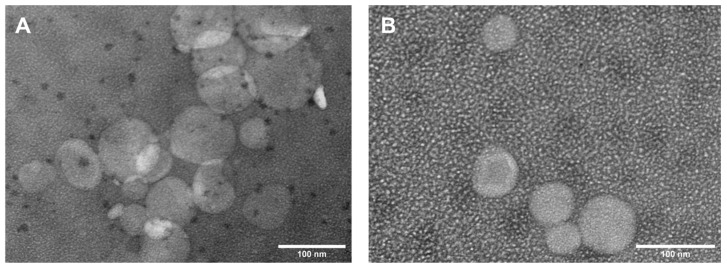
TEM images of blank liposomes (**A**) and sorafenib-functionalized liposomes (**B**).

Transmission electron microscopy was used to investigate the shape and the size of the liposomes. Typical TEM images of both classes of nanoliposomes are presented in Figure 3.

#### 3.1.4. Encapsulation Efficiency

Liposome drug loading was determined by HPLC-UV and was calculated using Equation (1). The encapsulation efficiency was estimated at 75.35% (±0.59).

### 3.2. In Vitro Evaluation of Liposomes on Cell Cultures

In vitro cytotoxicity effect of SOR, Lipo, and Lipo_SOR on normal human hepatic stellate cell line (LX-2), and two human hepatocarcinoma cell lines (SK-HEP-1 and HepG2), was evaluated in duplicates at 24 and 48 h after treatment. The cytotoxicity assays were performed for various concentrations of pure sorafenib SOR (2.5–20 µM) and sorafenib-encapsulated in liposomes Lipo_SOR (1.88–15.08 µM) in order to monitor the effects obtained by incorporating sorafenib in nanoliposomes. In the case of blank liposomes, their concentrations were adapted to the Lipo_SOR doses.

Data were processed using GraphPad Prism 6 software (log (concentration), data normalization, and nonlinear regression) and the IC50 concentrations of SOR, Lipo and Lipo_SOR determined for these cell lines are presented in Table 2.

The blank liposomes revealed no cytotoxicity effect when applied to the cell lines at both time points of the experiment.

**Figure 4:**
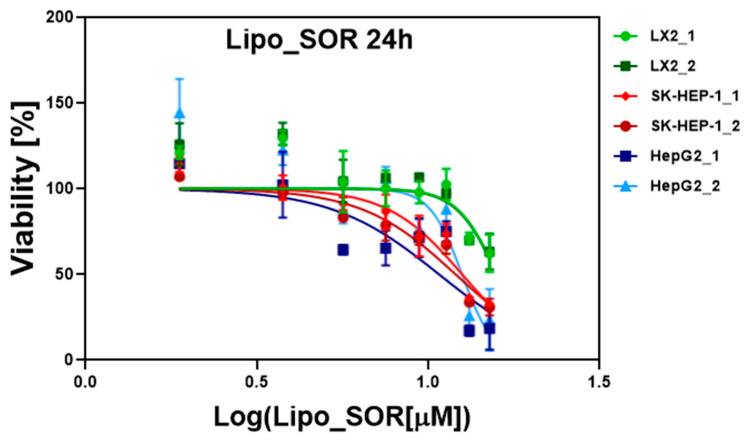
Evaluation of IC50 doses of Lipo_SOR treatment at 24 h. The experiments were performed in duplicates for each cell line. The SOR concentration was calculated based on EE values.

**Figure 5:**
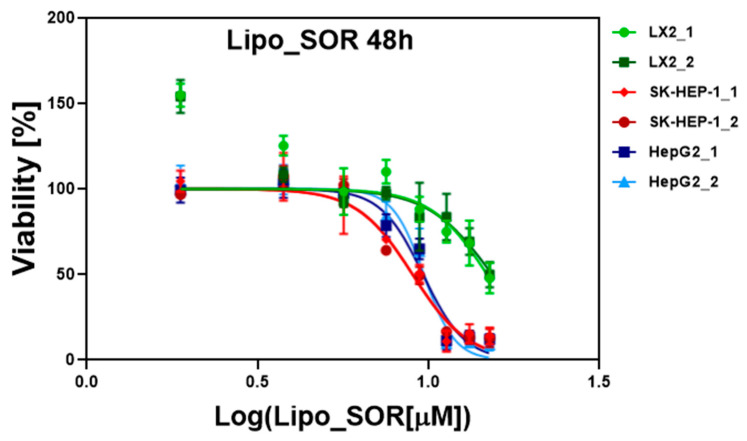
Evaluation of IC50 doses of Lipo_SOR treatment at 48 h. The experiments were performed in duplicates for each cell line. The SOR concentration was calculated based on EE values.

The IC50 graphs for Lipo_SOR at 24 and 48 h are presented in Figure 4 and Figure 5, while the other graphs, together with the statistical analysis for these experiments, are included in the Supplementary Materials (Figures S1–S4 and Tables S1–S3) attached to the manuscript.

A comparative analysis of the treatment effect at 24 h versus 48 h was performed. The IC50 results were analyzed using a t-test and the p-values together with standard deviations, and are summarized in Table 3. Since the blank liposomes do not affect cell viability, we chose to exclude their analysis.

## 4. Discussion

Our experiments allowed the synthesis of sorafenib-functionalized liposomes that were rigorously characterized. We also demonstrated their potential therapeutic efficacy on HCC by conducting in vitro studies on two types of cell cultures.

Raman spectroscopy was employed for the analysis of SOR, pure (unload-ed) liposomes, and sorafenib-loaded liposomes (Lipo_SOR) in order to prove that at the molecular level, sorafenib molecules have been inserted into the lipidic bilayer of the nanoliposomes.

The chemical structure of sorafenib includes one pyridine ring and two benzene rings. The quantum chemical studies performed on this molecule showed that the pyridine ring and one benzene ring are planar, while the second benzene ring is not planar. Stancioiu et al. have shown that the theoretical Raman spectra of sorafenib are dominated by a strong doublet located at 1599/1611 cm^−1^ that can be assigned to stretching vibrations of CC groups from the second benzene ring [37]. Another interesting peak is located at 370 cm^−1^ and it has been assigned to a combined vibration of the pyridine ring and benzene’s first ring. These two vibrational bands are highlighted in red in our spectrum from Figure 1. The spectrum is very similar to those reported in the scientific literature.

In the case of the liposomes, as can be seen in Figure 2, the Raman spectra of pure and loaded liposomes contain all the vibrational peaks characteristic of the three classes of lipids involved in liposomes’ synthesis: DSPC, DPPG, and Chol. This is clear evidence of the presence of these molecules in the composition of the liposomal membrane. The most important vibrational peaks are 718 and 785 cm^−1^ (assigned to different vibration modes of the choline group), and 1065, 1126, 1295, and 1439 cm^−1^ that can be assigned to vibrations of the CH, CH_2_, and CH_3_ groups.

The presence of the two vibrational peaks that have been mentioned before (375 and 1611 cm^−1^), assigned to specific vibrations of sorafenib, only in the case of Lipo_SOR, represents an irrefutable proof of sorafenib incorporation in the liposomal bilayer (red spectrum in Figure 2).

In the high wavenumber spectral region (2700–3100 cm^−1^), characteristics of carbon chain vibrations, three distinct peaks have been identified (2722, 2848, and 2876 cm^−1^). These peaks can be assigned to in-plane scissoring deformation vibrations and out-of-plane wagging deformation vibrations of the CH_2_ group. Upon the addition of sorafenib molecules to the bilayer, the intensity of all these peaks increases, suggesting a better organization of the lipid molecules, probably as a direct consequence of their interaction with sorafenib.

On the other hand, NTA analysis results indicate the presence of two major populations in both types of samples. In the case of Lipo_Blank, samples with 183 nm in diameter are predominant, while Lipo_SOR’s major population diameters are between 127–320 nm. These results were confirmed with TEM image analysis.

The drug encapsulation efficiency revealed a value of 75.35% according to the HPLC measurements. As highlighted by our experiments, the loading of sorafenib within liposomes allowed a passive accumulation of the drug within cells, leading to an enhanced therapeutic effect. This finding is supported by the IC50 values obtained in the case of cells treated with Lipo_SOR, as compared to those treated with pure SOR. This effect is even more pronounced in the case of tumoral cells, with respect to normal ones. In the case of the two HCC cell lines, the IC50 values decreased by ~30% at 24 h (12.25 vs. 16.93 µM for SK-HEP-1 and 11.61 vs. 16.95 µM for HepG2). For the 48-h treatment, this effect is more noticeable (~36% decrease in IC50 values for both tumoral cell lines), as can be seen in Table 2. In the case of normal cells (LX2) the decrease in IC50 doses is much lower (~16% for a 24-h and ~8% for a 48-h treatment), probably as a direct consequence of the fact that SOR therapeutic action is stronger for HCC cells, as compared to normal ones.

As it has been stated before, the IC50 values decreased at 48 h as compared to 24 h in the case of cancer cells. A comparative analysis of treatment with these IC50 values at 24 h versus 48 h has been performed. A p-value with high statistical significance has been obtained in the case of tumoral cells. Blank liposomes do not prove to have a cytotoxic effect on the analyzed cell lines, as expected.

However, the translation of our experiments into clinical practice should consider possible challenges considering the systemic or local drug delivery approach and the tumoral microenvironment. Although most nanoparticles tend to accumulate in the liver, the occurrence of HCC in cirrhotic tissue makes it a greater challenge than expected [3]. Selectively targeting tumoral hepatocytes defines another obstacle.

Evolving research has developed a broad range of nanoparticles for HCC based on alumina, arsenite, albumin, calcium, chitosan, gold, halifum oxide, iron oxide, lipids, poly(ethylene glycol) (PEG), platinum, poly(lactic-co-glycolic acid) (PLGA), polysaccharide, selenium, silica, silver, and zinc oxide [3]. We focused our research on liposomes, considering their structure of a single or multiple concentric lipid bilayers encapsulating an aqueous core, similar to the cellular membrane. They are biocompatible, biodegradable, nonimmunogenic, and have low toxicity. Their disadvantages consist of drug leakage, short half-life, possible oxidation, and hydrolysis of the used phospholipids [3].

Liposomes have been extensively studied as an approach to HCC treatment. Yang et al. prepared liposomal formulations entrapping docetaxel [38], demonstrating their efficacy on HCC cell lines. Further studies used different coated systems to increase the liposome’s stability and minimize its aggregation, cationic or anionic liposomes or pegylated ones; different combinations of toxic drugs showed promising results in vitro experiments [27,29,39,40,41,42,43,44,45,46,47,48]. Yin et al. formulated liposomes entrapping ceramides and sorafenib and showed a synergistic cytotoxic effect on HepG2 when compared to single drug liposomes [46].

To increase the efficacy of functionalized liposomes in vivo, ligands specific to the receptors on tumoral cells should be added (such as asialoglycoprotein receptor, glypican-3, transferrin receptor, folic acid receptor, and scavenger receptor class B type I) [3]. Further research is intended to increase the therapeutic potential of our functionalized liposomes.

Some research explored the effect of functionalized liposomes with cytotoxic drugs in combination with microwave ablation or transarterial chemoembolization [27,49]. Previous studies demonstrated that ultrasound in combination with microbubbles improved the delivery, local distribution, and therapeutic efficacy of drug-loaded nanoparticles in tumors [16,18]. This ultrasound effect, known as sonopermeation, applied to sorafenib-functionalized liposomes, is intended to be studied in our future research, as some of the treatment modalities of locally advanced HCC require ultrasound guidance.

## 5. Conclusions

Sorafenib-functionalized liposomes were synthesized and were rigorously characterized in order to evaluate the therapeutic effects through in vitro studies on cell cultures. Three hepatic cell lines were selected for these experiments, a normal LX2 cell line and two tumor cell lines, SK-HEP-1 and HepG2, respectively. The liposomes were synthesized using a rotary evaporator with a solvent evaporation method.

The physical properties of the liposomes were determined: in the case of simple liposomes, a homogeneous population with dimensions of up to 200 nm was obtained, and in the case of sorafenib-functionalized liposomes, medium-sized populations between 100 and 300 nm were predominant.

The TEM analysis of the two classes of nanoliposomes included in this study allowed the visualization of their spherical shape. The Raman analysis offered interesting nanoscale insights related to the interaction of SOR molecules with the lipids forming the hydrophobic liposomal bilayer. By analyzing the zeta-potential, slightly positive values were determined for both types of samples. Moreover, using the HPLC method, the encapsulation efficiency was determined.

SOR, Lipo, and Lipo_SOR were used for in vitro cell culture experiments, and their effects were evaluated after 24 and 48 h of treatment by the MTT cell viability test. In the case of hepatocarcinoma cell lines, it has been shown that the encapsulation of sorafenib in nanoliposomes leads to a strong decrease in the IC50 values as compared to those obtained in the case of the treatment with pure sorafenib, at both time points of the experiments (24 and 48 h).

## Figures and Tables

**Table 1:** The particle size, concentration, and zeta-potential of liposomes.

Sample	Particle Size (nm)	Percentage (%)	Concentration (NP/mL)	Zeta-Potential (mV)
Lipo	183	93.3	5.3 × 10^12^	5.05
	734	6.6		
Lipo_SOR	127–320	70.7	9.2 × 10^10^	11.29
	724–960	29.3		

**Table 2:** IC50 concentrations (µM) of SOR, Lipo, and Lipo_SOR.

Treatment_Time	LX2 Cells	SK-HEP-1 Cells	HepG2 Cells
SOR_24 h	18.695	16.935	16.95
SOR_48 h	16.3	13.935	15.145
Lipo_24 h	-	-	-
Lipo_48 h	-	-	-
Lipo_SOR_24 h	15.76	12.25	11.615
Lipo_SOR_48 h	15.02	8.96	9.63

**Table 3:** Statistical analysis of the IC50 concentrations’ results.

Cell Lines	Lipo_SOR 24 vs. 48 h	SOR 24 vs. 48 h
LX2	*p* = 0.1 ^ns^	*p* = 0.11 ^ns^
	SD_24_ = 0.021	SD_24_ = 1.237
	SD_48_ = 0.361	SD_48_ = 0.099
SK-HEP-1	*p* = 0.006 **	*p* = 0.007 **
	SD_24_ = 0.382	SD_24_ = 0.290
	SD_48_ = 0.002	SD_48_ = 0.205
HepG2	*p* = 0.154 (ns)	*p* = 0.017 *
	SD_24_ = 1.252	SD_24_ = 0.212
	SD_48_ = 0.033	SD_48_ = 0.262

SD_24_, SD_48_—standard deviations at 24 h and 48 h, respectively; ns—non significant; *—*p* ≤ 0.05; **—*p* ≤ 0.01.

## Data Availability

Not applicable.

## Supplementary Materials

The following supporting information can be downloaded at: https://www.mdpi.com/article/10.3390/nano12162833/s1, Figure S1: Evaluation of IC_50_ doses of Lipo treatment at 24 h. The experiments were performed in duplicates for each cell line; Figure S2: Evaluation of IC_50_ doses of Lipo at 48 h. The experiments were performed in duplicates for each cell line; Figure S3: Evaluation of IC_50_ doses of SOR treatment at 24 h. The experiments were performed in duplicates for each cell line; Figure S4: Evaluation of IC_50_ doses of SOR treatment at 48 h. The experiments were performed in duplicates for each cell line; Table S1: Statistical analysis in the case of blank liposomes treatments effect on 24 and 48 h performed in duplicates, Table S2: Statistical analysis in the case of sorafenib treatments effect on 24 and 48 h performed in duplicates, Table S3: Statistical analysis in the case of sorafenib-encapsulated liposomes treatments effect on 24 and 48 h performed in duplicates.
