# Supplementary material for: Hybrid Lipid Nanoformulations for Hepatoma Therapy: Sorafenib Loaded Nanoliposomes—A Preliminary Study
Source: Nanomaterials (Basel). 2022 Aug 17;12(16):2833. doi: 10.3390/nano12162833 (PMC9414144; doi:10.3390/nano12162833)
Supplement: Supplementary file 1 [file nanomaterials-12-02833-s001.zip › nanomaterials-1814296-supplementary.pdf]

nanomaterials

Article

Hybrid Lipid Nanoformulations for Hepatoma Therapy: Sorafenib Loaded Nanoliposomes—A Preliminary Study

Adrian Bartos [1,2], Ioana Iancu [3,*], Lidia Ciobanu [1], Anca Onaciu [4,5], Cristian Moldovan [4,5], Alin Moldovan [4], Radu Cristian Moldovan [4], Adrian Bogdan Tigu [4], Gabriela Fabiola Stiufiuc [6], Valentin Toma [4], Cornel Iancu [1], Nadim Al Hajjar [1,2] and Rares Ionut Stiufiuc [4,5,*]

1 Department of Surgery, Regional Institute of Gastroenterology and Hepatology, 400162 Cluj-Napoca, Romania
2 Department of Surgery, "Iuliu Hatieganu" University of Medicine and Pharmacy, 400337 Cluj-Napoca, Romania
3 Department of Surgery, Medicover Hospital, 407062 Cluj-Napoca, Romania
4 MedFuture—Research Center for Advanced Medicine, "Iuliu Hatieganu" University of Medicine and Pharmacy, 400337 Cluj-Napoca, Romania
5 Department of Pharmaceutical Physics-Biophysics, "Iuliu Hatieganu" University of Medicine and Pharmacy, 400349 Cluj-Napoca, Romania
6 Faculty of Physics, "Babes Bolyai" University, 400084 Cluj-Napoca, Romania
* Correspondence: iancuioana2011@gmail.com (I.I.); rares.stiufiuc@umfcluj.ro (R.I.S.); Tel.: +40-747-795-612 (I.I.); +40-726-340-278 (R.I.S.)

Citation: Bartos, A.; Iancu, I.; Ciobanu, L.; Onaciu, A.; Moldovan, C.; Moldovan, A.; Moldovan, R.C.; Tigu, A.B.; Stiufiuc, G.F.; Toma, V.; et al. Hybrid Lipid Nanoformulations for Hepatoma Therapy: Sorafenib Loaded Nanoliposomes—A Preliminary Study. *Nanomaterials* **2022**, *12*, 2833. https://doi.org/10.3390/nano12162833

Academic Editor: Zili Sideratou

Received: 28 June 2022
Accepted: 11 August 2022
Published: 17 August 2022

Publisher's Note: MDPI stays neutral with regard to jurisdictional claims in published maps and institutional affiliations.

Copyright: © 2022 by the authors. Submitted for possible open access publication under the terms and conditions of the Creative Commons Attribution (CC BY) license (https://creativecommons.org/licenses/by/4.0/).

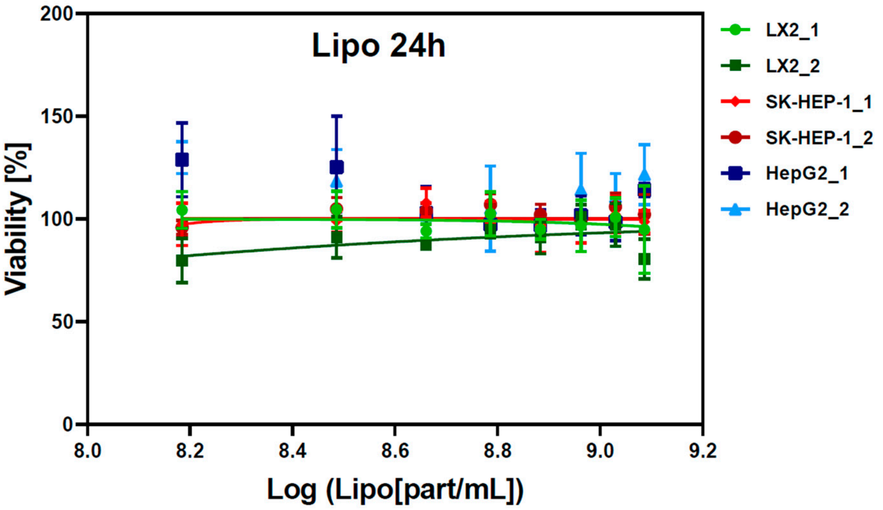

Figure S1. Evaluation of IC_{50} doses of Lipo treatment at 24 h. The experiments were performed on duplicate for each cell line.

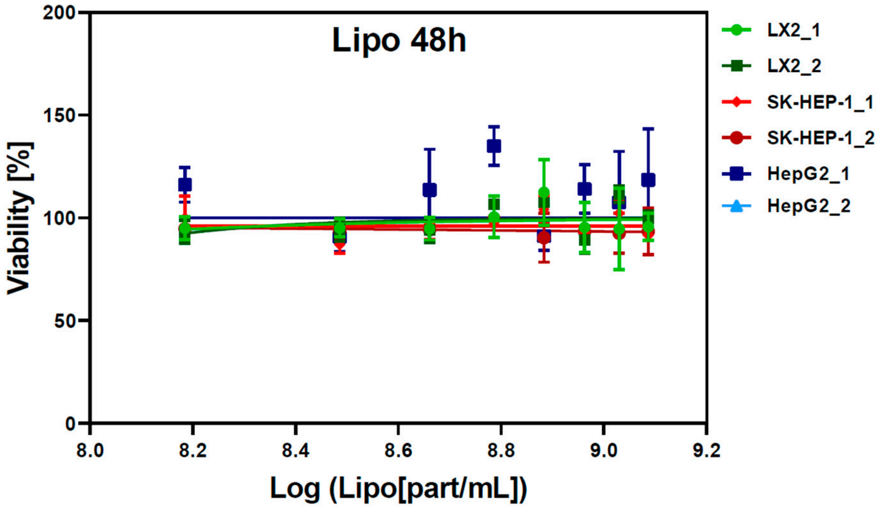

Figure S2. Evaluation of IC50 doses of Lipo at 48 h. The experiments were performed on duplicate for each cell line.

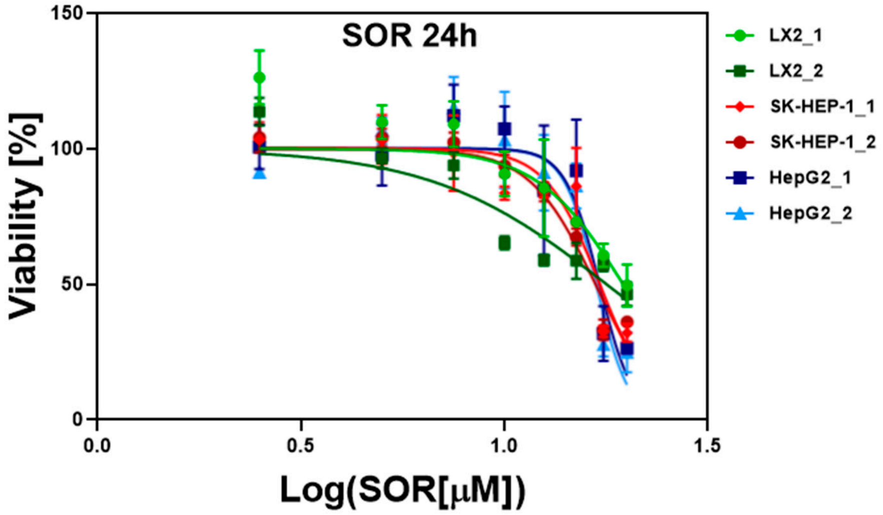

Figure S3. Evaluation of IC50 doses of SOR treatment at 24 h. The experiments were performed on duplicate for each cell line.

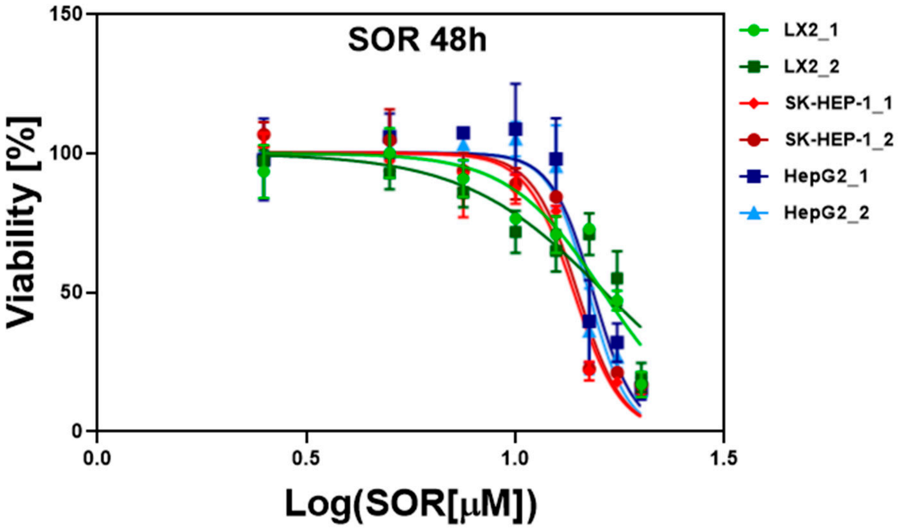

Figure S4. Evaluation of IC_{50} doses of SOR treatment at 48 h. The experiments were performed on duplicate for each cell line.

Table S1. Statistical analysis in the case of blank liposomes treatments effect on 24 and 48 h.

Cell line	1.22E+09 part/ml	1.07E+09 part/ml	9.17E+08 part/ml	7.64E+08 part/ml	6.11E+08 part/ml	4.58E+08 part/ml	3.06E+08 part/ml	1.53E+08 part/ml
Duplicate 1 24h								
LX2	0.769328	0.947867	0.817012	0.678590	0.849069	0.624289	0.723553	0.735599
SKHEP	0.772832	0.730028	0.857338	0.107944	0.458241	0.179640	0.795779	0.702046
HEPG2	0.045177	0.848654	0.810619	0.648596	0.588334	0.761715	0.167473	0.063622
Duplicate 2 24h								
LX2	0.087038	0.765906	0.898290	0.308474	0.419783	0.126129	0.365206	0.088683
SKHEP	0.757732	0.328753	0.715451	0.695232	0.219940	0.411216	0.367465	0.375057
HEPG2	0.172483	0.422020	0.380560	0.833452	0.755598	0.860321	0.235949	0.050281
Duplicate 1 48h								
LX2	0.438823	0.677189	0.584409	0.268253	0.946938	0.310058	0.293018	0.321055
SKHEP	0.024743 ↑*	0.621539	0.043293 ↑*	0.417953	0.272675	0.094648	0.018794 ↑*	0.990909
HEPG2	0.256268	0.294356	0.710103	0.971164	0.009092 ↑**	0.947129	0.564198	0.023731 ↑*
Duplicate 2 48h								
LX2	0.087038	0.765906	0.898290	0.308474	0.419783	0.126129	0.365206	0.088683
SKHEP	0.388417	0.276780	0.054595	0.244687	0.852371	0.053357	0.111586	0.048279 ↑*
HEPG2	0.172483	0.422020	0.380560	0.833452	0.755598	0.860321	0.235949	0.050281

↑ - increased viability; ↓ - decreased viability; P-value <0.05 (*), P-value <0.01 (**) and P-value <0.001 (***).
Discovery determined using the Two-stage linear step-up procedure of Benjamini, Krieger and Yekutieli, with Q = 1%.
Each row was analyzed individually, without assuming a consistent SD. Number of t tests: 3.

Table S2. Statistical analysis in the case of Sorafenib treatments effect on 24 and 48 h.

Cell line	20 µM	17.5 µM	15 µM	12.5 µM	10 µM	7.5 µM	5 µM	2.5 µM
Duplicate 1 24h								
LX2	0.001208 ↓***	0.001287 ↓***	0.003413 ↓***	0.263599	0.218253	0.229341	0.151472	0.021292 ↓*
SKHEP	0.000308 ↓***	0.000279 ↓***	0.233513	0.050174	0.044889 ↓*	0.874839	0.675029	0.580075
HEPG2	0.000591 ↓***	0.001958 ↓***	0.570509	0.381779	0.452142	0.288447	0.827872	0.943004
Duplicate 2 24h								
LX2	0.000302 ↓***	0.000493 ↓***	0.001641 ↓**	0.000520 ↓**	0.001062 ↓**	0.263530	0.459452	0.048789 ↓*
SKHEP	0.000005 ↓***	0.000017 ↓***	0.000171 ↓***	0.003403 ↓**	0.089980	0.423007	0.171031	0.309791
HEPG2	0.000413 ↓***	0.000283 ↓***	0.130263	0.416992	0.799905	0.159974	0.339079	0.193787
Duplicate 1 48h								
LX2	0.000064 ↓***	0.000289 ↓***	0.002609 ↓**	0.006094 ↓**	0.004276 ↓**	0.176328	0.991945	0.390000
SKHEP	0.000017 ↓***	0.000021 ↓***	0.000038 ↓***	0.004603 ↓**	0.048843 ↓*	0.057974	0.472784	0.634548
HEPG2	0.000108↓***	0.000454↓***	0.003706↓**	0.840712	0.466975	0.221270	0.437520	0.830644
Duplicate 2 48h								
LX2	0.000302 ↓***	0.000493 ↓***	0.001641 ↓**	0.000520 ↓***	0.001062 ↓**	0.263530	0.459452	0.048789
SKHEP	0.000005 ↓***	0.000017 ↓***	0.000171 ↓***	0.003403 ↓**	0.089980	0.423007	0.171031	0.309791
HEPG2	0.000413 ↓***	0.000283 ↓***	0.130263	0.416992	0.799905	0.159974	0.339079	0.193787

↑ - increased viability; ↓ - decreased viability; P-value <0.05 (*), P-value <0.01 (**) and P-value <0.001 (***).
Discovery determined using the Two-stage linear step-up procedure of Benjamini, Krieger and Yekutieli, with Q = 1%.
Each row was analyzed individually, without assuming a consistent SD. Number of t tests: 3.

Table S3. Statistical analysis in the case of Lipo_SOR treatments effect on 24 and 48 h.

Cell line	15.08 µM	13.17 µM	11.28 µM	9.4 µM	7.51 µM	5.64 µM	3.76 µM	1.88 µM
Duplicate 1 24h								
LX2	0.004120 ↓***	0.000160 ↓***	0.672048	0.612151	0.984074	0.714449	0.000332 ↑***	0.000898 ↑***
SKHEP	0.000004 ↓***	0.000006 ↓***	0.002297 ↓**	0.018093 ↓*	0.093159	0.038076 ↓*	0.903695	0.011109 ↑*
HEPG2	0.005756 ↓**	0.003551 ↓**	0.145049	0.127007	0.076598	0.057279	0.901877	0.361602
Duplicate 2 24h								
LX2	0.007062 ↓**	0.001828 ↓**	0.562223	0.182374	0.177440	0.610755	0.003986 ↑**	0.034356 ↑*
SKHEP	0.000048 ↓***	0.000018 ↓***	0.001027 ↓**	0.000934 ↓***	0.021367 ↓*	0.002733 ↓**	0.554501	0.066765
HEPG2	0.002364 ↓**	0.000267 ↓***	0.146928	0.010949 ↓*	0.348438	0.101571	0.026474↑*	0.021574 ↑*
Duplicate 1 48h								
LX2	0.001312 ↓***	0.020901 ↓*	0.010244 ↓*	0.112281	0.146689	0.874352	0.008119 ↑**	0.000603 ↑***
SKHEP	0.000036 ↓***	0.000048 ↓***	0.000050 ↓***	0.000347↓***	0.000917 ↓***	0.408659	0.464314	0.393643
HEPG2	0.000127 ↓***	0.000143 ↓***	0.000176 ↓***	0.006473 ↓**	0.036219 ↓*	0.685431	0.704974	0.930465
Duplicate 2 48h								
LX2	0.013747 ↓*	0.062985	0.295600	0.428195	0.835431	0.569662	0.505756	0.012286 ↑*
SKHEP	0.000028 ↓***	0.000015 ↓***	0.000010 ↓***	0.000220 ↓***	0.000260 ↓***	0.548100	0.167830	0.342118
HEPG2	0.000096 ↓***	0.000087 ↓***	0.000082 ↓***	0.018249 ↓*	0.097682	0.912583	0.502474	0.586861

↑ - increased viability; ↓ - decreased viability; P-value <0.05 (*), P-value <0.01 (**) and P-value <0.001 (***).
Discovery determined using the Two-stage linear step-up procedure of Benjamini, Krieger and Yekutieli, with Q = 1%.
Each row was analyzed individually, without assuming a consistent SD. Number of t tests: 3.
